# Identification of protein methyltransferases 5 associated with ferroptosis and immune cell infiltration of head and neck squamous cell carcinoma

**DOI:** 10.18632/aging.205768

**Published:** 2024-04-25

**Authors:** Xiaoyue Zhang, Qiang Liu, Lujuan Wang, Qiu Peng

**Affiliations:** 1 Hunan Cancer Hospital and The Affiliated Cancer Hospital of Xiangya School of Medicine, Central South University, Changsha, China; 2 Department of Orthopaedics, The Second Xiangya Hospital of Central South University, Changsha, Hunan, China; 3 Hunan Key Laboratory of Tumor Models and Individualized Medicine, The Second Xiangya Hospital of Central South University, Changsha, Hunan, China; 4 Cancer Research Institute and School of Basic Medical Science, Central South University, Changsha, China; 5 The Second Department of Breast Surgery, Hunan Cancer Hospital, The Affiliated Cancer Hospital of Xiangya School of Medicine, Central South University, Changsha, China

**Keywords:** head and neck tumors, PRMT

## Abstract

Head and neck tumors are malignant tumors that appear in the head and neck. Although much progress has been made in the treatment of head and neck tumors, many challenges remain. The prognosis of some advanced cases remains poor and survival and quality of life after treatment face certain limitations. Therefore, further research into the pathogenesis and treatment options for head and neck tumors is important in order to improve the prognosis and quality of life of patients. The Protein Arginine Methyltransferase (PRMT) family is a class of enzymes that are responsible for adding methyl groups to arginine residues in proteins. PRMT family members play important roles in regulating many cellular processes, such as transcriptional regulation, signaling, and cell cycle regulation. Recent studies have shown that the PRMT family also plays an important function in tumorigenesis and development. Here, we found that PRMT family members are significantly overexpressed in head and neck tumors and that PRMT5 may serve as an independent prognostic factor in head and neck tumors. We found that PRMT5-regulated differential genes were significantly enriched in tumor-associated signaling pathways such as IL-17 and p53. And we also found that the expression of PRMT5 in head and neck tumors was significantly correlated with immune cell infiltration, m6A as well as the expression of ferroptosis-related genes, and drug sensitivity. These results suggest that PRMT may play an important role in the development of head and neck tumors.

## INTRODUCTION

Head and neck tumors (HNSC) are malignant tumors that appear in the head and neck. They can occur in the mouth, throat, tongue, vocal cords, buccal cavity, nasal cavity, and thyroid gland [1]. HNSC are one of the common malignant tumors worldwide and have a significant impact on the quality of life and survival of patients. The causes of HNSC are complex, including genetic factors, environmental factors, and viral infections. Smoking and alcohol abuse are one of the main causes of head and neck tumors, while infection with human papillomavirus (HPV) is also closely related to the development of HNSC [2]. In addition, factors such as long-term exposure to harmful substances, chronic inflammation, and malnutrition may also increase the risk of head and neck tumors. HNSC have a variety of clinical manifestations, including neck lumps, dysphagia, hoarseness, and mouth ulcers. Early diagnosis and treatment are important to improve the survival rate and quality of life of patients. Common diagnostic methods include history inquiry, physical examination, imaging examination, tissue biopsy, etc. [3]. For the treatment of HNSC, early cases can usually be cured by surgical removal of the tumor, but advanced cases may require the combined use of radiation therapy and chemotherapy [4]. In addition, immunotherapy and targeted therapy have been widely studied for the treatment of HNSC [5, 6].

PRMT is a class of arginine methyltransferases. PRMT family members play important roles in regulating many cellular processes, such as transcriptional regulation, signaling, and cell cycle regulation [7, 8]. In tumors, aberrant expression of PRMT family is usually associated with tumorigenesis, progression and prognosis [9, 10]. Several studies have found that overexpression of PRMT1, PRMT3, and PRMT6 is associated with increased progression and invasiveness of many tumor types [11–14]. These PRMT family members can regulate the expression of tumor-associated genes, thereby promoting tumor cell proliferation, migration, and invasion. On the other hand, some studies have also found that the expression levels of PRMT2 and PRMT5 are down-regulated in tumors, and their functions are associated with inhibition of tumor cell proliferation and invasion [15, 16]. This suggests that different PRMT family members may have different roles in tumors. In addition to the abnormal expression of PRMT family members, the regulation of their activities and functions may also have important effects on tumorigenesis and progression. It was found that some members of the PRMT family were found to interact with tumor-associated proteins, regulate their methylation status, and further affect the function of these proteins [17, 18]. This implies that it is possible to develop therapeutic strategies for targeting tumors by modulating the activity and function of PRMT family members.

Here, we found that PRMT family members are significantly overexpressed in HNSC and that PRMT5 may serve as an independent prognostic factor in HNSC. We found that PRMT5-regulated differential genes were significantly enriched in tumor-associated signaling pathways such as IL-17 and p53. And we also found that the expression of PRMT5 in HNSC was significantly correlated with immune cell infiltration, m6A as well as the expression of ferroptosis-related genes, and drug sensitivity. In summary, the PRMT family plays an important role in tumorigenesis and development, and its abnormal expression and activity regulation are closely related to the expression of tumor-related genes and cellular functions. So in-depth study of the function and regulatory mechanism of PRMT family in HNSC is of great significance to understand the mechanism of tumorigenesis and development, and to provide new targets and strategies for tumor diagnosis and treatment.

## RESULTS

### Expression and mutation of PRMT in HNSC

**Figure 1:**
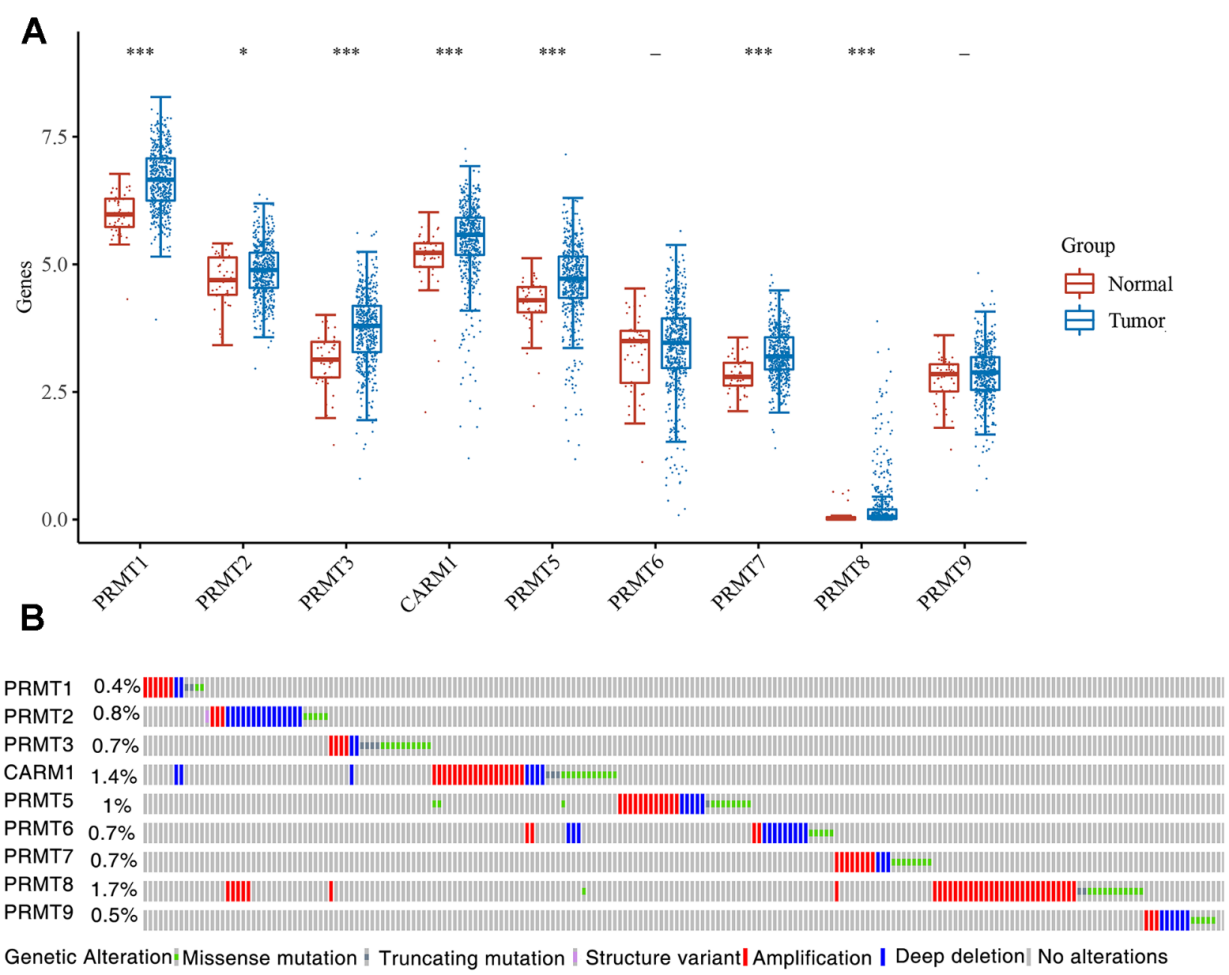
**Expression and TMB of PRMT in HNSC.** (**A**) The expression distribution of PRMT in tumor tissues and normal tissues. (**B**) Mutation frequency of the PRMT in HNSC. *p < 0.05, **p < 0.01, ***p < 0.001.

To investigate the role of PRMT in the development of HNSC, we first analyzed the expression levels of PRMT family members (PRMT1-PRMT9) in tumors using TCGA data, and we found that PRMT6 and PRMT9, the other PRMT family members, had significantly elevated expression in tumor tissues (Figure 1A). We know that tumor development is ultimately caused by mutations in genes, and we further analyzed the mutations in PRMT in HNSC and found that overall, the frequency of mutations in PRMT is not high, with only PRMT4 also called CARM1 (1.4%), PRMT 5 (1%) and PRMT 8 (1.7%) having a relative mutation frequency greater than 1% (Figure 1B).

### Confirmation of the prognostic relationship between PRMT and HNSC

**Figure 2:**
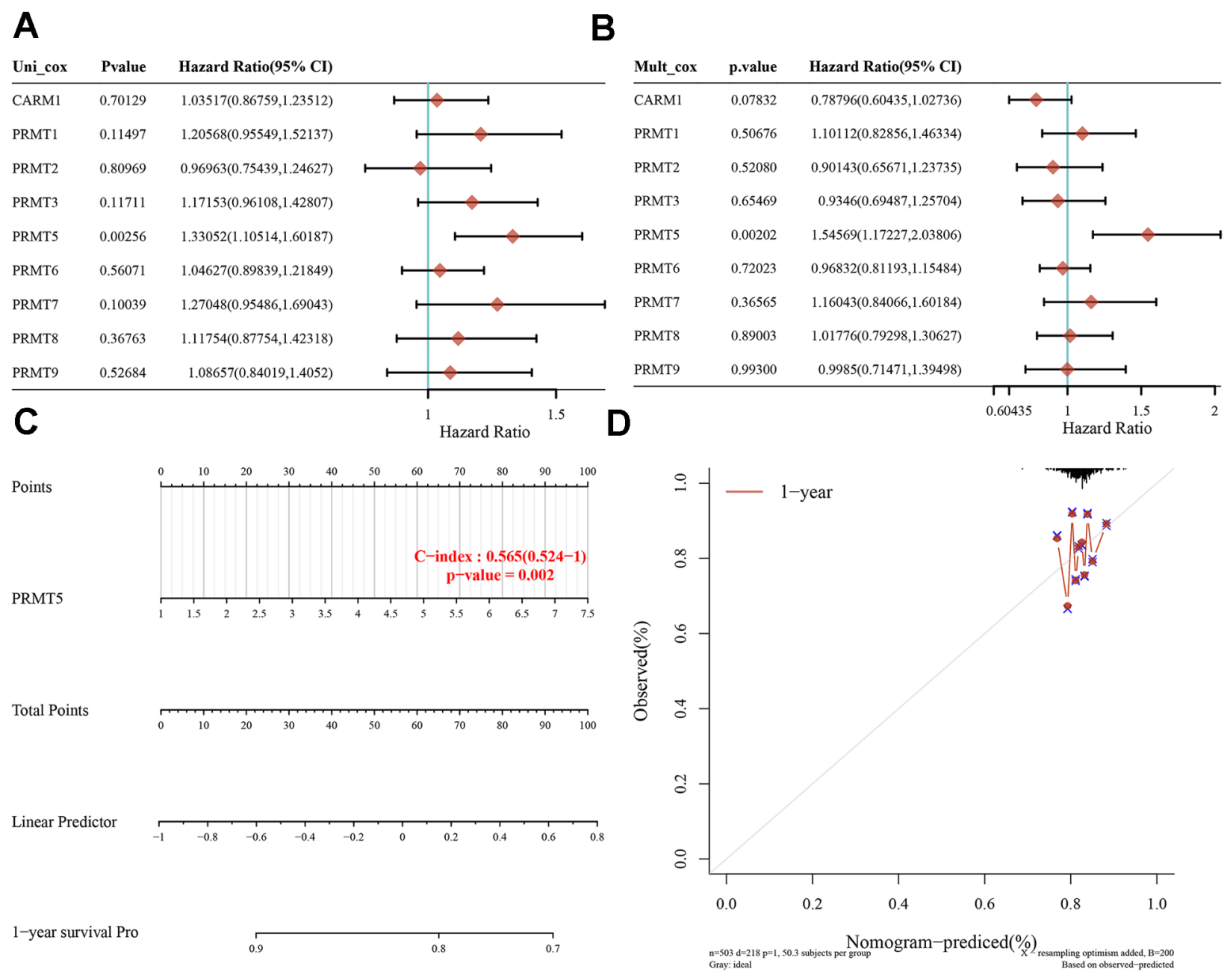
**PRMT expression correlates with the prognosis of patients with HNSC.** (**A**, **B**) Univariate and multivariate Cox regression analyses of the relationship between PRMT family and prognosis of HNSC. (**C**, **D**) Nomogram predicts 1-year overall survival for HNSC patient.

In order to analyze the effect of PRMT on the prognosis of HNSC, we first found by univariate and multivariate regression analysis that only PRMT5 was statistically significant in both methods of analysis (Figure 2A, 2B). Based on the results of a multivariate Cox proportional risk analysis, a column chart was constructed using the “rms” package to predict the overall 1-year recurrence rate. PRMT5 was found to be a risk factor affecting the prognostic risk of patients. PRMT5 was found to be a risk factor influencing patient prognosis (Figure 2C, 2D). These findings suggest that PRMT5 can predict patient survival in HNSC and that high PRMT5 expression is associated with poor prognosis in HNSC.

### PRMT5-regulated signaling pathways

**Figure 3:**
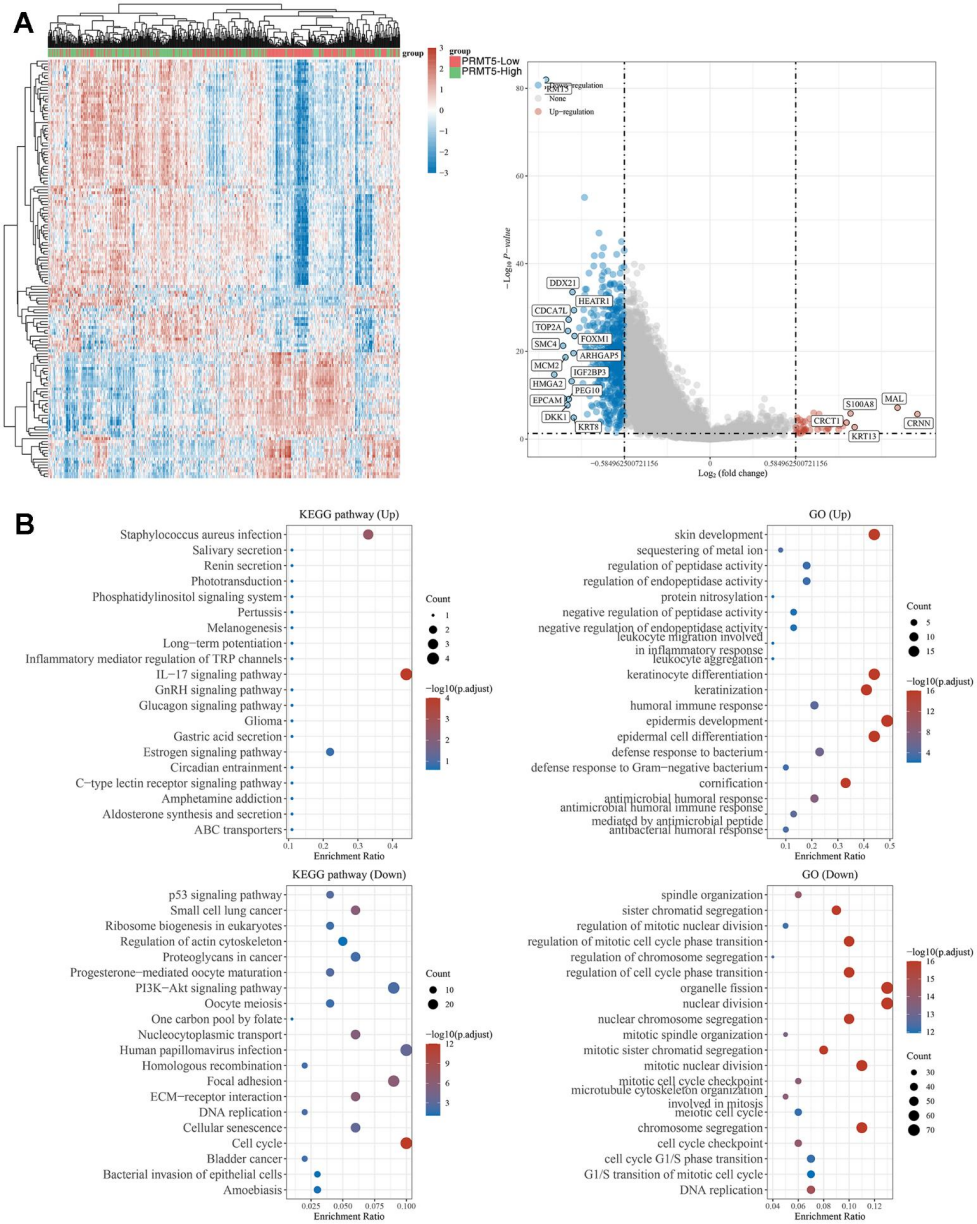
**Biological functions of PRMT5 in HNSC samples.** (**A**) Heat map and Venn diagram showing differentially expressed genes in HNSC with high and low expression of PRMT5. (**B**) KEGG and GO analyses of PRMT5-regulated differentially expressed genes in HNSC.

To explore the mechanisms by which PRMT5 may regulate the development of head and neck tumor, we first analysed differential gene expression in samples with differential PRMT5 expression using TCGA HNSC samples, and in total we identified a large number of PRMT5-regulated genes, including DDX21, S100A8, SMC4 (Figure 3A). We further performed KEGG and GO analysis on the differential genes regulated by PRMT5 and found that these gene-enriched pathways are closely related to tumor development, such as IL-17, P53, PI3K-AKT and other signaling pathways (Figure 3B). These results suggest that PRMT5 may promote the progression of HNSC by activating tumor-associated signaling pathways.

### Correlation of PRMT5 expression with immune cell infiltration and immune checkpoint expression

**Figure 4:**
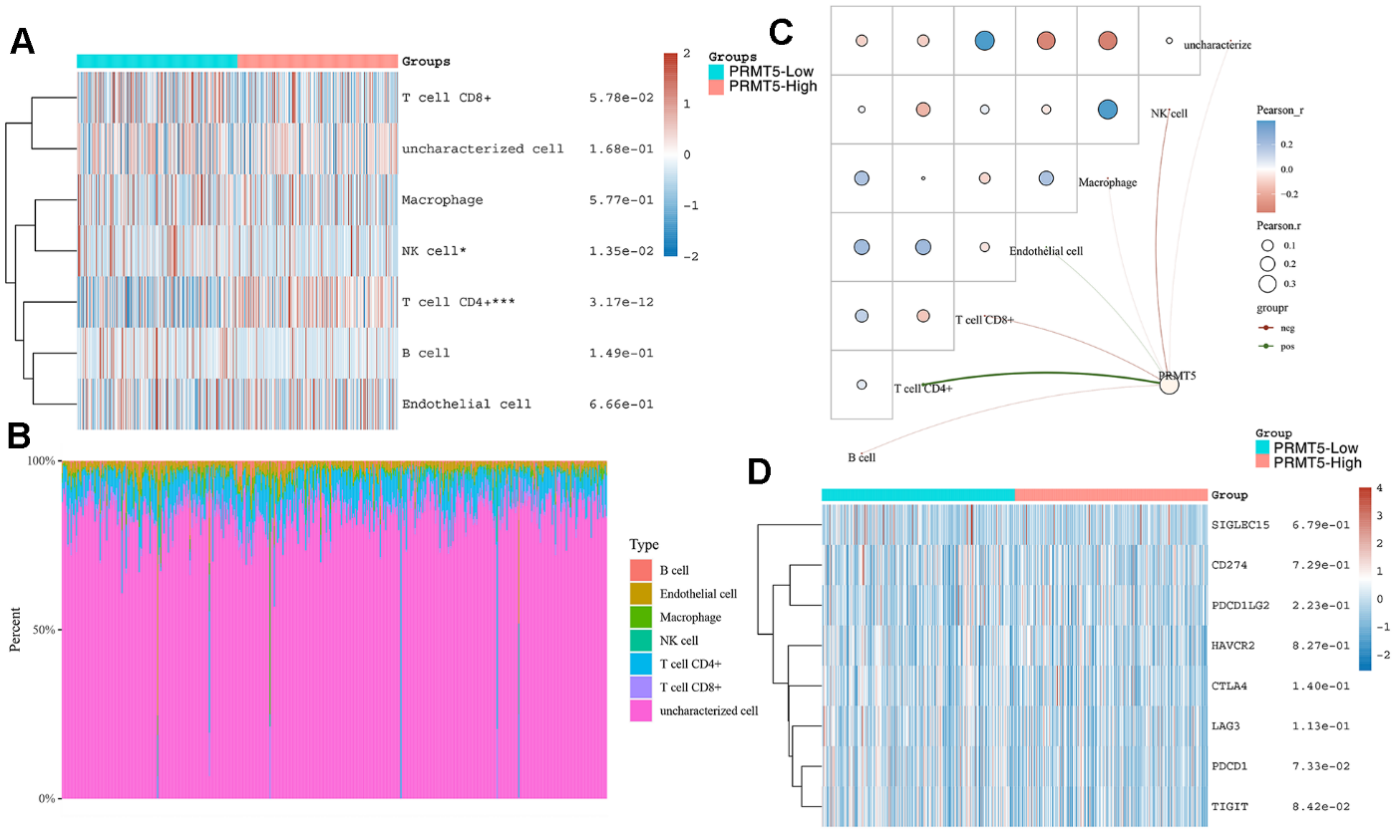
**Correlation between PRMT5 expression and various immune cells infiltration and expression distribution of immune checkpoint.** (**A**–**C**) The immune score and (**D**) immune-checkpoint-related gene expression and PRMT5 expression. Each box in the figure represents the correlation analysis between the expression of the PRMT5 and the immune score and immune checkpoint in corresponding tumors. *p < 0.05, **p < 0.01, ***p < 0.001.

Tumor immunotherapy is an emerging approach to tumor treatment that utilizes the patient’s own immune system to identify, attack and remove tumor cells. The goal of immunotherapy is to increase the body’s immune response to tumors in order to achieve long-term control or even elimination of tumors [19]. To investigate the relationship between PRMT5 expression and immune cell infiltration in HNSC, we found that PRMT5 expression showed a significant positive correlation with NK cells as well as CD4-positive T-cell infiltration by TIMER algorithm using TCGA data (Figure 4A–4C). We also further analyzed the correlation between PRMT5 expression and common immune checkpoints in HNSC, including, SIGLEC15, TIGIT, CD274, HAVCR2, PDCD1, CTLA4, LAG3 and PDCD1LG2 etc. We found no significant correlation between PRMT5 expression and the expression of common immune checkpoints (Figure 4D). These results suggest that PRMT5 may affect the efficacy of immunotherapy mainly by influencing the infiltration of immune cells, but not by influencing the expression of immune checkpoints.

### Correlation analysis of PRMT5 expression with m6A-related gene expression

**Figure 5:**
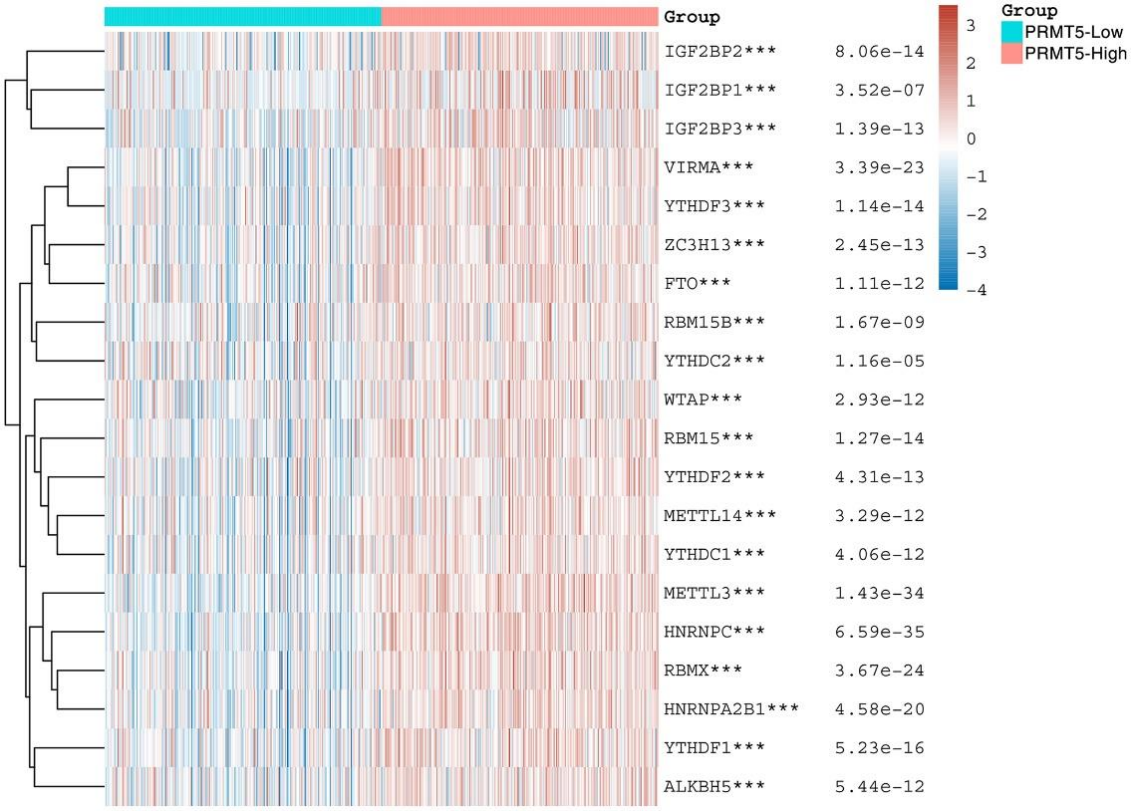
**PRMT5 is associated with m6A modification.** Heat map showing the correlation of PRMT5 expression with the expression of common m6A-related genes. *stands for significance levels, *for p < 0.05, **p < 0.01, ***p < 0.001.

N6-methyladenosine (m6A) is a common RNA modification that plays an important role in a variety of biological processes, including transcriptional regulation, RNA splicing, RNA stability and translation. Recent studies have shown that m6A modification also plays an important regulatory role in tumorigenesis and development [20]. In order to investigate the correlation between the expression of PRMT5 and the expression of m6A-related genes in HNSC, we analyzed the TCGA data and found that there was a significant positive correlation between the expression of PRMT5 and the m6A-related genes (Figure 5), which suggests that PRMT5 may regulate the expression of m6A-related genes and thus promote the development of HNSC.

### Correlation analysis of PRMT5 expression and ferroptosis-related genes in HNSC

**Figure 6:**
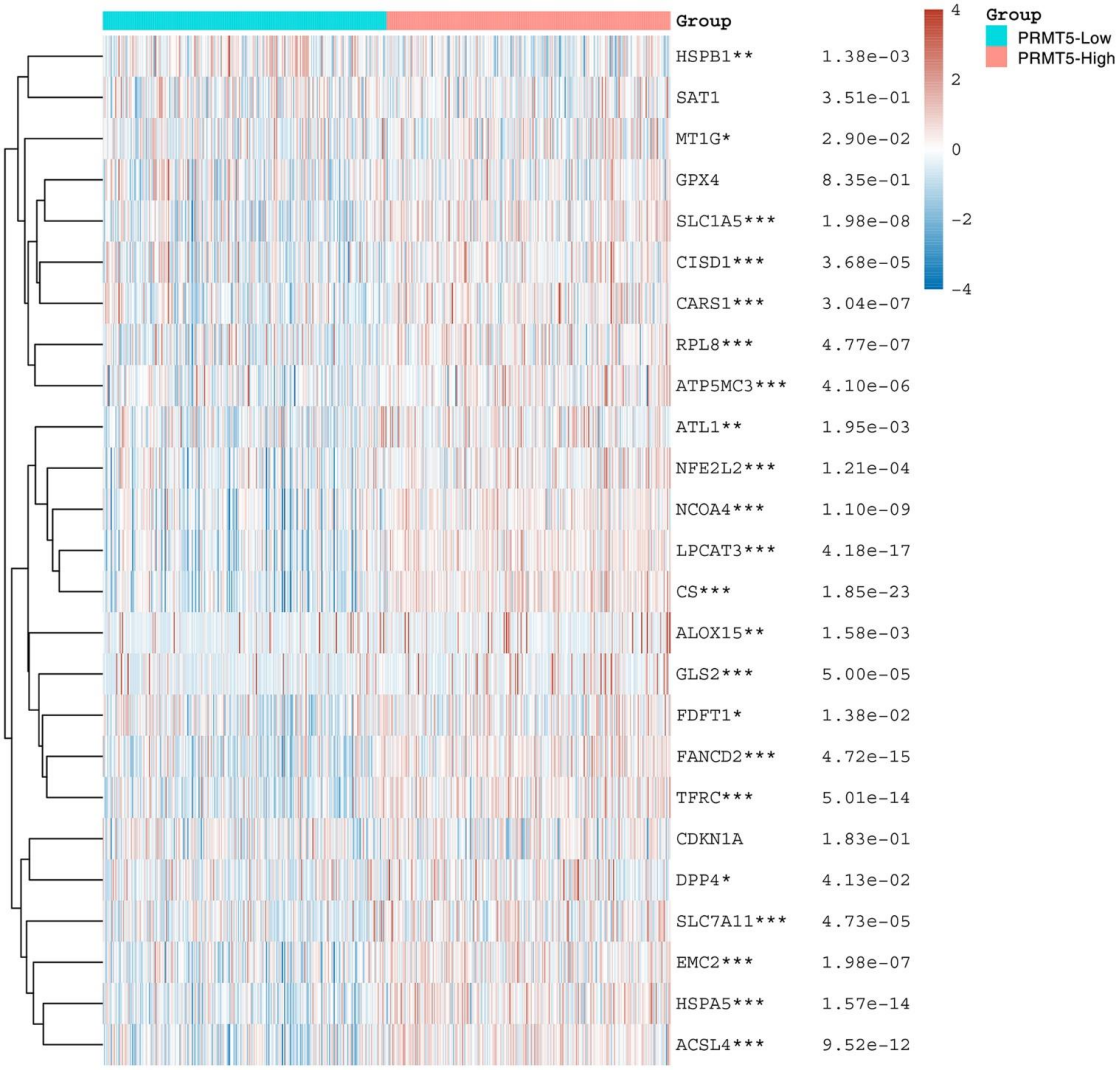
**PRMT5 is associated with ferroptosis.** Heat map showing the correlation of PRMT5 expression with the expression of common ferroptosis-related genes. *stands for significance levels, *for p < 0.05, **p < 0.01, ***p < 0.001.

Ferroptosis is a newly discovered mode of cell death that is different from traditional apoptosis, necrosis and autophagy. Studies have shown that Fe ions play an important role in regulating cell death. In tumors, ferroptosis has also been suggested as a possible important influence on tumorigenesis and progression [21]. To explore the correlation between PRMT5 expression and the expression of ferroptosis -related genes, we analyzed the data using TCGA and found that the expression of PRMT5 showed significant correlation with the vast majority of ferroptosis-related genes, except for GPX4, SAT1, and CDKN1A (Figure 6). This suggests that PRMT5 may influence the progression of head and neck cancer by regulating ferroptosis.

### Expression of PRMT5 is correlated with drug sensitivity

**Figure 7:**
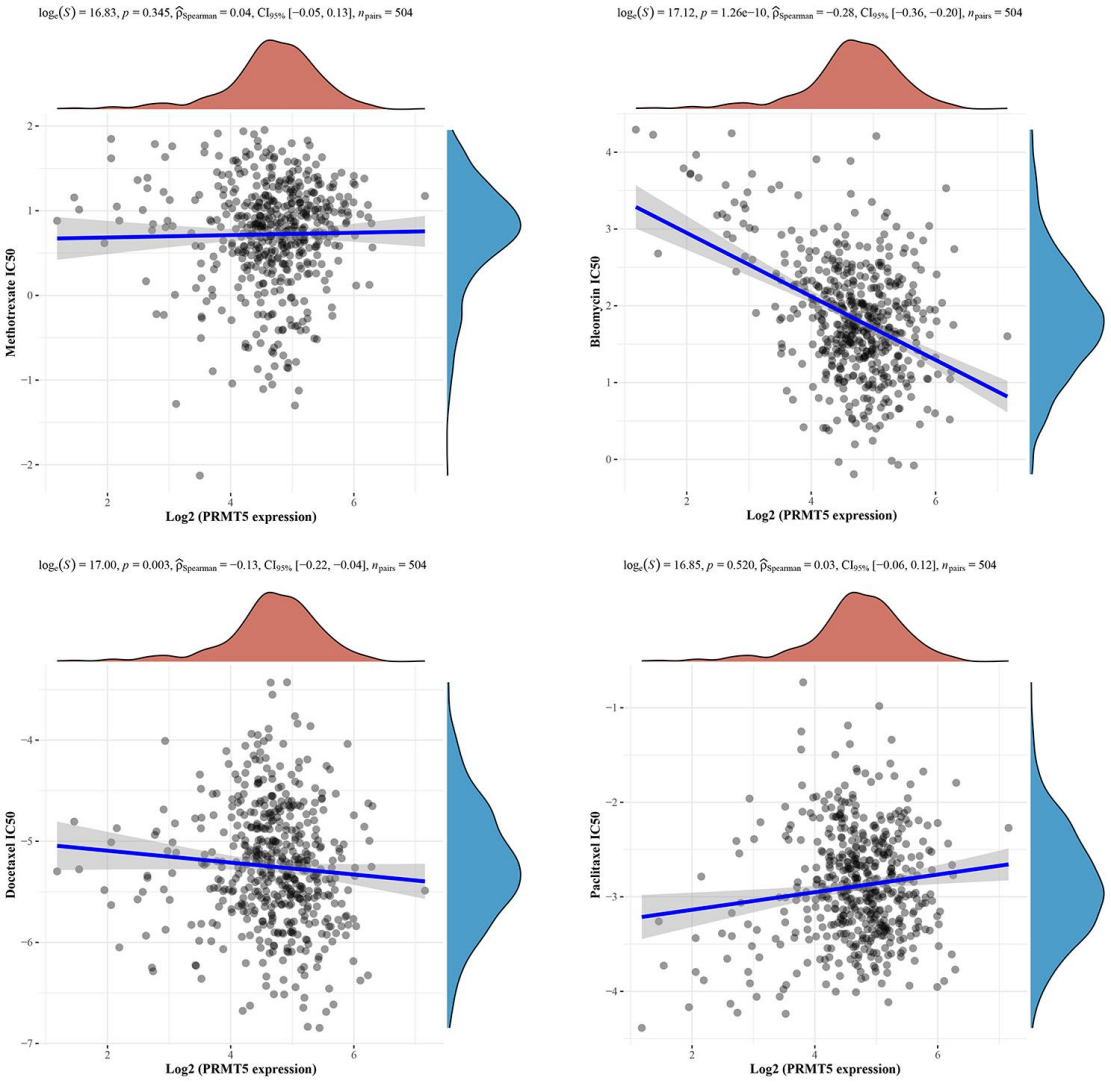
**Correlation between PRMT5 expression and drug sensitivity.** Spearman correlation analysis of methotrexate, bleomycin, docetaxel and paclitaxel IC50 score and PRMT5 expression.

Drug sensitivity is of great importance in tumor therapy. By evaluating the sensitivity of tumor cells to drugs, it can provide guidance for individualized treatment regimens so that the most suitable drug or treatment strategy for the patient can be selected. Optimized drug selection not only improves therapeutic efficacy, but also reduces the occurrence of unwanted side effects and drug resistance. In order to investigate the correlation between PRMT5 expression and sensitivity to common therapeutic agents for HNSC, including agents methotrexate, bleomycin, docetaxel and paclitaxel, we found that PRMT5 expression was significantly and positively correlated with IC50 in methotrexate and paclitaxel, while it was significantly and negatively correlated with bleomycin and docetaxel using the GDPC tool (https://www.cancerrxgene.org/) (Figure 7), which suggests that we need to make some differences in the administration of drugs for patients with different PRMT5 expression.

## DISCUSSION

Head and neck squamous cell carcinoma is a common malignant tumor of the head and neck that originates from epithelial cells [1]. It occurs mainly in the oral cavity, pharynx, larynx, and esophagus, and is usually associated with risk factors such as smoking, alcohol abuse, and human papillomavirus (HPV) infection [22]. Head and neck squamous cell carcinoma is usually characterized by aggressive growth and early metastasis, affecting patients’ quality of life and survival. Early diagnosis is particularly important for the treatment of head and neck squamous cell carcinoma. Treatment strategies usually include surgical resection, radiotherapy, chemotherapy and targeted therapy. In recent years, the application of immunotherapy has also brought new hope for the treatment of head and neck squamous cell carcinoma [5, 23]. Despite some challenges in treatment, early prevention, early diagnosis and individualized treatment remain important strategies for managing head and neck squamous cell carcinoma. In this study, we found that PRMT family members are significantly overexpressed in HNSC and that PRMT5 may serve as an independent prognostic factor in HNSC. We found that PRMT5-regulated differential genes were significantly enriched in tumor-associated signaling pathways such as IL-17 and p53. And we also found that the expression of PRMT5 in HNSC was significantly correlated with immune cell infiltration, m6A as well as the expression of ferroptosis-related genes, and drug sensitivity. So in-depth study of the function and regulatory mechanism of PRMT family in HNSC is of great significance to understand the mechanism of tumorigenesis and development, and to provide new targets and strategies for tumor diagnosis and treatment.

It has been found that the pattern and level of m6A modification in tumors are usually different from that in normal tissues. aberrant expression or disorders of m6A modification have been associated with a wide range of tumors, including lung, breast, bladder, gastric, liver, and colorectal cancers. This suggests that m6A modification may have an important regulatory role in tumorigenesis and development [24, 25]. m6A modifications can regulate tumor biological processes in several ways. First, m6A modifications can affect the proliferation, invasion and metastatic ability of tumor cells. Second, m6A modifications can regulate the stem cell properties and drug resistance of tumor cells [26, 27]. In addition, m6A modifications can affect the immune escape of tumors and the formation of the tumor microenvironment. The m6A modifications are mainly regulated by three core enzymes (“Writer”) and various “Readers” and “Erasers”. The “Writers” include methyltransferases such as METTL3, METTL14 and WTAP, which are responsible for adding m6A modifications to RNA molecules. The “Readers” include m6A recognition proteins such as YTHDF1, YTHDF2, YTHDF3, YTHDC1, and HNRNPA2B1, which are able to recognize and bind RNAs with m6A modifications. The “Eraser” includes two proteins: ALKBH5 and FTO, whose main role is to remove the m6A modification [28]. In our study, we found a significant positive correlation between PRMT5 and the expression of m6A-related genes, suggesting that in HNSC PRMT5 may influence the development of HNSC by regulating the expression of m6A-related genes. Despite the initial understanding of the role of m6A modification in tumors, there are still many unknowns. For example, the specific mechanisms of m6A modification, regulatory networks and interactions with other regulatory factors still require further research. Further studies will help to reveal the mechanism of the role of m6A modification in tumorigenesis and development and provide a theoretical basis for the development of new therapeutic strategies.

In recent years, through the development of high-throughput technologies, researchers have begun to utilize large-scale data such as genomes, transcriptomes, and proteomes to study the prediction of drug sensitivity. These studies have constructed predictive models by analyzing data from multiple dimensions, such as gene expression profiles, mutation profiles, and copy number variations of tumor samples, to help understand the mechanisms involved in drug sensitivity and predict patient response [29, 30]. In our study, we also found that PRMT5 expression was associated with different drug sensitivities with different benefits, which suggests that there is a need for some differentiation in dosing for HNSC patients with different expression of PRMT5. Although some progress has been made in drug sensitivity research, many challenges remain. Heterogeneity among different tumor types and patients, complex molecular regulatory networks in tumor cells, and dynamic changes during tumor progression add to the complexity of predicting drug sensitivity. Therefore, continued in-depth research into the mechanisms and prediction methods of drug sensitivity can help to more accurately predict patient response and guide the development of individualized tumor treatment strategies.

## MATERIALS AND METHODS

### Expression and TMB analysis of PRMT

Expression profiling data and TMB for HNSC as well as normal samples were obtained using The Cancer Genome Atlas (TCGA) database (https://portal.gdc.cancer.gov), and bioinformatics was used to analyze the differences in PRMT expression in all tumor samples and normal tissues.

### Immune cell infiltration and immune checkpoint analysis

RNA-sequencing expression profiles and corresponding clinical information for HNSC were downloaded from the TCGA dataset. To assess the reliable results of immune score evaluation, we used immuneeconv. It’s an R software package that integrates TIMER algorithms.

### Drug sensitivity analysis

RNA-sequencing expression profiles and corresponding clinical information for HNSC were downloaded from the TCGA dataset. Predicted the chemotherapeutic response for each sample based on the largest publicly available pharmacogenomics database [the Genomics of Drug Sensitivity in Cancer (GDSC), https://www.cancerrxgene.org/]. The prediction process was implemented by R package “pRRophetic”. The samples’ half-maximal inhibitory concentration (IC50) was estimated by ridge regression. All parameters were set as the default values. Using the batch effect of combat and tissue type of all tissues, and the duplicate gene expression was summarized as mean value.
